# Nurse-Led Interventions Targeting Clinical Correlates of Immunosenescence in Older Adults: A Scoping Review

**DOI:** 10.3390/medicina62020262

**Published:** 2026-01-26

**Authors:** Gianluca Azzellino, Patrizia Vagnarelli, Ernesto Aitella, Luca Mengoli, Lia Ginaldi, Massimo De Martinis

**Affiliations:** 1 Department of Life, Health and Environmental Sciences, University of L’Aquila, Piazzale Salvatore Tommasi n. 1, 67100 L’Aquila, Italy; ernesto.aitella@graduate.univaq.it (E.A.); lia.ginaldi@univaq.it (L.G.); 2 Complex Operational Unit, Adriatic District Area, AUSL 04 Teramo, 64100 Teramo, Italy; patrizia.vagnarelli@aslteramo.it; 3 Allergy and Clinical Immunology Unit, Center for the Diagnosis and Treatment of Osteoporosis, AUSL 04 Teramo, 64100 Teramo, Italy; 4 Long-Term Care Unit, “Maria SS. Dello Splendore” Hospital, AUSL 04 Teramo, 64021 Giulianova, Italy; luca.mengoli@aslteramo.it; 5 UniCamillus-Saint Camillus International University of Health Sciences, 00131 Rome, Italy

**Keywords:** primary care nursing, community nursing, older adults, frailty, frail elderly, nurse-led interventions, immunosenescence, aged

## Abstract

*Background and Objectives:* Immunosenescence is a complex biological process associated with aging, characterized by a progressive decline in immune function and increased chronic inflammation (“inflammaging”), with clinical implications such as frailty, functional decline, multimorbidity, and a higher risk of adverse events in older adults. Nurses in community and primary care settings play a central role in preventive and health promotion interventions that may indirectly influence these processes. However, the available literature remains fragmented. Therefore, this scoping review aims to map and synthesise nursing interventions targeting older adults (≥60 years) that may indirectly influence immunosenescence by acting on its clinical correlates and modifiable determinants, organising the evidence within a four-pillar conceptual framework. *Materials and Methods:* A scoping review was conducted following JBI methodology and the PRISMA-ScR checklist. We included primary studies on nurse-led interventions in community, home care, primary care, territorial, or long-term care settings. PubMed, Scopus, and Web of Science were searched (English; last 10 years). Interventions were classified into four pillars: nursing nutrition and immunonutrition support, physical activity and exercise support, nursing vaccination coaching, and frailty monitoring and prevention of functional decline. *Results:* Twenty-five primary studies were included, mostly randomised or cluster-randomised trials in community, primary care, home care, and transitional care settings. Interventions mapped mainly to Pillar 4 and Pillar 2, while Pillar 1 was less frequent and usually part of multicomponent programmes; no primary studies targeted Pillar 3. Overall, effectiveness appeared driven more by intervention intensity and integration than by frailty identification alone: structured, multicomponent nurse-led programmes combining exercise with nutritional and psychosocial components showed the most consistent benefits on frailty, functional outcomes, and well-being, whereas low-intensity preventive consultations and Comprehensive Geriatric Assessment (CGA)-based models often showed limited improvements over usual care. *Conclusions:* This scoping review highlights the key role of community and primary care nurses in preventive interventions targeting clinical correlates of immunosenescence. Multicomponent nurse-led programmes integrating physical activity, nutrition, and psychosocial support appear most promising for frailty and functional outcomes, while low-intensity interventions show limited effectiveness. No primary studies addressed nurse-led vaccination coaching, representing an evidence gap. Future research should include biological/immunological markers alongside clinical outcomes.

## 1. Introduction

**Figure 1:**
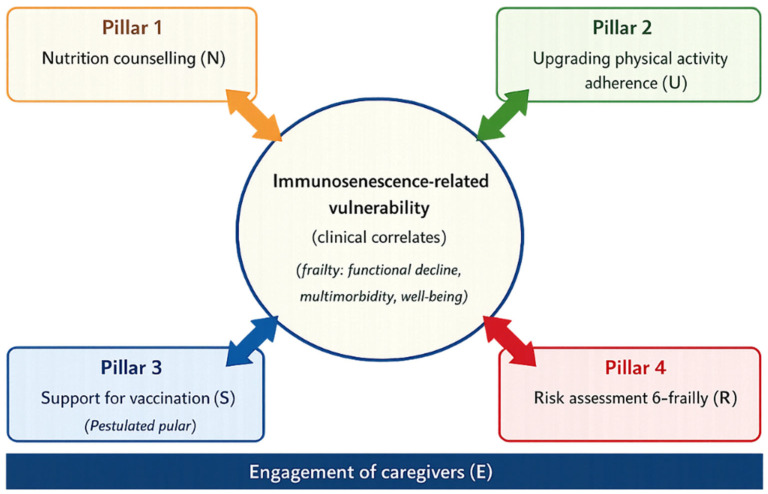
The NURSE AGE heuristic framework integrating nurse-led interventions across four pillars, with caregiver engagement as a cross-cutting component, to address immunosenescence-related clinical correlates and guide future research.

Population ageing represents a major challenge for healthcare systems worldwide. Life expectancy is projected to exceed 80 years by 2040, leading to a growing proportion of the population exposed to immunosenescence, inflammaging, and frailty [1,2]. This demographic shift is associated with a higher prevalence of chronic conditions, multimorbidity, and frailty, with significant implications for health outcomes and the organization of healthcare services [2,3]. Moreover, these conditions increase older adults’ vulnerability to both endogenous and environmental stressors [4]. Among the main biological processes associated with ageing, immunosenescence, defined as the progressive and multifactorial deterioration of the immune system linked to advancing age [5], represents a key determinant of increased susceptibility to infections, reduced vaccine responsiveness, and progressive functional decline [5,6]. This is further supported by evidence on vaccine safety and immunogenicity in clinically vulnerable populations [7], and immunosenescence is also characterised by immune cell dysfunction and apoptosis remodelling [8]. This process is closely associated with inflammaging, a state of chronic low-grade inflammation driven by immuno-endocrine and cytokine alterations, which contributes to the development of clinical frailty and sarcopenia and represents a shared pathogenic mechanism across multiple age-related conditions, thereby increasing the risk of hospitalization and mortality in older adults [9,10,11,12]. Recent evidence suggests that immunosenescence should not be regarded as a strictly irreversible process, but rather as a biological trajectory that may be modulated through lifestyle interventions and other modifiable health determinants, as well as through the regulation of chronic inflammatory pathways and immuno-neuro-endocrine interactions associated with ageing [13,14,15]. In particular, nutrition, physical activity, vaccination strategies, and frailty monitoring are closely linked to the biological mechanisms of immune ageing, including through structured behavioral interventions delivered in primary care settings [16,17,18]. Low-intensity nutritional interventions, when integrated into community nursing practice, may further support functional and immune status among older adults living at home within primary care and community nursing contexts [18]. In this context, community and primary care nurses represent a strategic resource, particularly to strengthen prevention, early detection, and outreach strategies among underserved and marginalised populations [19] as highlighted during the COVID-19 pandemic, with evidence suggesting that SARS-CoV-2 may have been circulating in Italy already in early December 2019 [20]. Nurse-led or community nurse-guided programs have combined education, counseling, follow-up, and multidimensional interventions with the aim of preventing or mitigating frailty and improving health outcomes among older adults in home-based settings, including proactive models of screening, multidimensional assessment, and personalized interventions delivered in primary care and community settings [21,22,23,24]. Through these activities, nursing care may support health-related behaviors, treatment adherence, and the management of risk factors associated with immunosenescence [22]. Despite their clinical relevance, these interventions are described in a fragmented and heterogeneous manner, lacking a unified conceptual framework [25]. In light of these considerations, this scoping review aims to map the available evidence on nurse-led, nurse-delivered, or nurse-involved interventions implemented in community nursing, home care, and primary care settings and targeting older adults (≥60 years) that may potentially influence clinical and functional correlates of immunosenescence. Interventions are organized within four pillars: nursing nutrition and immunonutrition support, support for physical activity, vaccination coaching, and frailty monitoring with preventive interventions. Finally, based on the evidence identified, a heuristic conceptual framework, the NURSE AGE Framework (Nutrition counseling, Upgrading physical activity adherence, Risk assessment for frailty, Support for vaccination, Engagement of caregivers), is proposed as an expert synthesis to integrate findings across the four pillars, support the interpretation of potential links with immunosenescence, identify gaps in the literature, and outline practical implications and directions for future research, including testing this framework in future intervention studies (Figure 1). This framework was not inductively derived from an empirical synthesis of effect estimates, but is proposed as a conceptual model to generate hypotheses and guide future research.

## 2. Methods

This study is a scoping review conducted following the JBI methodology [26] and reported according to the PRISMA-ScR checklist [27]. The review protocol was not prospectively registered on any platform (e.g., PROSPERO, Open Science Framework). This does not affect the aims of the present scoping review, which was designed to map the available evidence rather than to synthesise intervention effects. Nevertheless, the review methods were developed a priori in accordance with the JBI framework and PRISMA-ScR guidance to ensure methodological rigour and transparency. The research question guiding the review was:

What nursing-led interventions have been described in the literature as potentially capable of influencing clinical correlates of immunosenescence in older adults?

To address this question, the PCC framework was applied [26]:

- Population: Older adults aged ≥ 60 years, including frail older adults and individuals with multimorbidity living in community or long-term care settings.
- Concept: Nursing-led or nursing-delivered interventions potentially influencing immune function or its clinical correlates (e.g., inflammaging, frailty, functional decline, vaccine response).
- Context: Community and territorial care settings, including community nursing, home care nursing, primary care, and integrated home care services.

### 2.1. Search Strategy and Databases

The search strategy was developed using natural language keywords combined with Boolean operators to identify nurse-led interventions targeting older adults in community and primary care settings, as well as domains potentially associated with immunosenescence and its clinical correlates. In this review, community and primary care refers to healthcare services delivered in community-based, home care, or primary care contexts. Terms related to acute hospital care, critical care, or intensive care units were excluded; however, studies conducted in mixed settings were eligible when the intervention targeted older adults in community contexts. Searches were conducted in PubMed, Scopus, and Web of Science, limited to English-language publications from the last 10 years to capture contemporary nurse-led models and ensure relevance to current organisational and preventive care frameworks. Grey literature and manual reference list screening were not performed. The final search was completed on 23 December 2025. The full search strategies are reported in Supplementary File S1.

### 2.2. Study Selection and Screening Process

**Figure 2:**
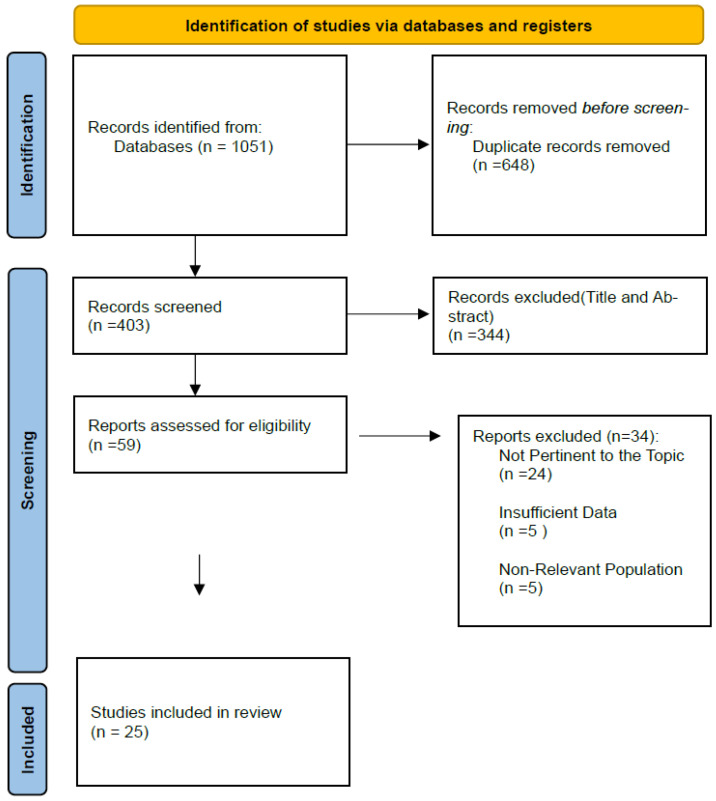
PRISMA-ScR flow diagram illustrating the study selection process. The databases searched were PubMed, Scopus, and Web of Science.

Study selection was performed in two phases. First, two reviewers (GA and MD) independently screened titles and abstracts against the predefined inclusion and exclusion criteria, excluding clearly non-eligible records. Second, the full texts of potentially relevant studies were independently assessed to confirm eligibility. Records were managed and duplicates removed using Zotero (Corporation for Digital Scholarship, Vienna, VA, USA; version 7.0.21). Disagreements at any stage were resolved through discussion and consensus, with the involvement of a third reviewer (GL) when necessary. The selection process followed PRISMA-ScR recommendations and is illustrated in Figure 2.

### 2.3. Inclusion and Exclusion Criteria

The inclusion and exclusion criteria were defined a priori based on the objectives of the scoping review and the four-pillar conceptual model adopted for the analysis of nursing interventions.

#### 2.3.1. Inclusion Criteria

Studies were included if they met all of the following criteria:

- Population: Adults aged ≥ 60 years, including frail older adults and individuals with multimorbidity.
- Intervention: Studies describing nursing-led or nursing-delivered interventions potentially capable of modulating immunosenescence or its clinical correlates, such as inflammaging, frailty, functional decline, or vaccine response. Explicit reference to the term *immunosenescence* was not mandatory, provided that the intervention could be mapped to at least one of the four pillars of the conceptual framework.
- Care setting: Community and territorial care settings, including community nursing, home care nursing, primary care, integrated home care services, and residential or long-term care facilities.
- Nursing role: Studies in which the intervention was nurse-led or nurse-delivered, with an active and central role of nurses in the design and/or delivery of the intervention.
- Study design: Primary studies, including experimental and quasi-experimental studies, observational studies with an interventional component, and pilot studies.
- Language and publication period: Studies published in English within the last 10 years.

#### 2.3.2. Exclusion Criteria

Studies were excluded if they:

- did not describe a nurse-led or nurse-delivered intervention (e.g., interventions delivered exclusively by other professionals);
- were conducted exclusively in acute hospital, intensive care, or critical care settings with no community/primary care component;
- were secondary studies (systematic reviews, scoping reviews, narrative reviews), editorials, commentaries, or protocols;
- were purely descriptive qualitative studies without an intervention component; qualitative studies were eligible only if they described and evaluated a nurse-led intervention (e.g., feasibility, acceptability, or participants’ experiences);
- did not allow mapping of the intervention to at least one of the four pillars.

### 2.4. Data Extraction

Data extraction was performed independently by two reviewers (GA and MD) using a standardized extraction form, developed in accordance with the JBI guidelines for scoping reviews [26]. The extraction form was designed to ensure consistency with the objectives of the review and with the four-pillar conceptual model, and included the following variables: author and year of publication, study design, care setting, reference conceptual pillar(s), description of the nurse-led or nurse-delivered nursing intervention, main outcomes and key findings. To ensure the accuracy, completeness, and consistency of the extracted data, a third reviewer (PV) verified all collected information. Any discrepancies or uncertainties that arose during the extraction process were discussed among the reviewers and resolved by consensus.

### 2.5. Data Synthesis

The results were summarized using a narrative approach. Data extracted from the included studies were initially organized in a structured table and subsequently sorted according to the four-pillar conceptual model, defined a priori. Specifically, each study was assigned to one of the four pillars based on the primary focus of the nursing intervention described:

(1) nursing nutrition and immunonutrition support (nutritional risk monitoring and nurse-led nutritional counselling),
(2) physical activity and exercise support,
(3) nursing vaccination coaching,
(4) frailty monitoring and prevention of functional decline.

For each pillar, the results were summarized in narrative form describing the study design, the care setting, the type of nurse-led nursing intervention, the main outcomes assessed, and the key results, as reported in the data extraction table.

## 3. Results

A total of 1051 records were identified through database searching. After removing 648 duplicates, 403 titles and abstracts were screened, and 344 records were excluded. Fifty-nine full-text articles were assessed for eligibility, of which 34 were excluded. Finally, 25 studies were included in the review (Figure 2).

### 3.1. Selection and General Characteristics of the Included Studies

The included studies (n = 25) are heterogeneous in terms of methodological design, intensity of interventions and care settings, with most located in community care and primary care. Nursing interventions (nurse-led or nurse-delivered) were analysed according to four predefined conceptual pillars: (1) nursing nutrition and immunonutrition support, (2) physical activity and exercise support, (3) nursing vaccination coaching, and (4) frailty monitoring and prevention of functional decline. Randomised controlled trials (including cluster RCTs) predominate among study designs and are frequently used for frailty prevention programmes, physical activity promotion and post-discharge interventions (e.g., Bleijenberg et al. [24]; Bleijenberg et al. [28]; Dorresteijn et al. [29]; Harris et al. [30]; Suijker et al. [31]; Suijker et al. [32]; Verloo et al. [33]; Xu et al. [34]; Xu et al. [35]; Sönmez Sari & Kitiş [36]). Quasi-experimental and pre-post studies are common in community settings (senior centres, local services), as documented by Lee et al. [37], Song & Boo [38], Kang et al. [39] and Marcus-Varwijk et al. [40]. Observational and mixed-methods approaches focus on service utilisation, hospital–community continuity, and clinical and psychosocial outcomes (e.g., Tam et al. [41]; Ron et al. [42]), while a single qualitative study explores a “low-threshold” nutritional intervention based on meal sharing (Frøyland Alne et al. [18]).

#### Care Settings

The care settings of the included studies show a clear prevalence of community care, primary care and home care, which represent the main setting for the nursing interventions mapped. There are also post-hospitalisation transitional care programmes, outpatient interventions and integrated hospital-community models, as documented by van der Vlegel-Brouwer et al. [43], Tam et al. [41] and Ron et al. [42]. The main characteristics of the included studies are summarised in Table 1.

### 3.2. Main Results

**Figure 3:**
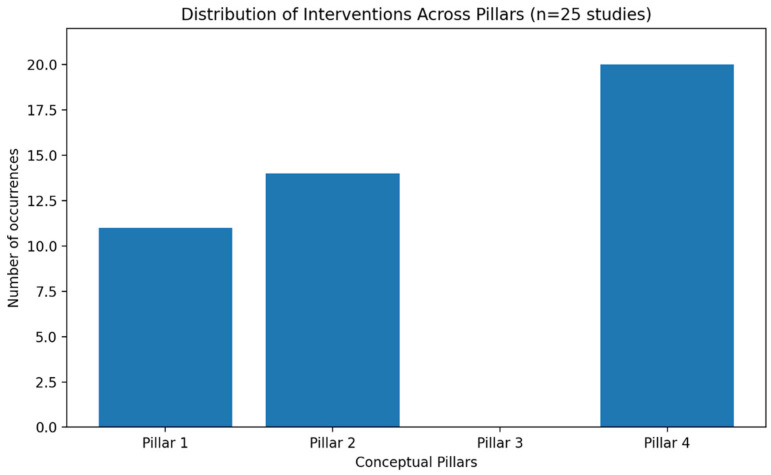
Frequency of nursing interventions mapped to the four conceptual pillars.

Overall, the included studies described nurse-led interventions delivered through multiple modalities (home visits, group sessions, individual counselling, telephone follow-ups, and digital support), which were mainly mapped to Pillar 2 (physical activity and exercise support) and Pillar 4 (frailty monitoring and prevention of functional decline). A smaller number of interventions include components attributable to Pillar 1 (nursing nutrition and immunonutrition support), most often embedded within multi-component or multifactorial programmes. No primary empirical studies were identified for Pillar 3 (nursing vaccination coaching); therefore, it was not possible to map results for this pillar. As shown in Figure 3, nursing interventions were predominantly mapped to Pillar 4 (frailty monitoring and prevention of functional decline), followed by Pillar 2 (physical activity and exercise support).

#### Results Summarised by Framework Pillars

- **Pillar 1—Nutritional risk monitoring and nurse-led nutritional counselling**

Interventions included in this pillar comprised nutritional screening, nurse-led education and counselling, practical support with food intake, and follow-up, sometimes embedded within multidimensional programmes. In rural settings, nurse-led interventions based on structured nutritional assessment (MNA) and personalised recommendations were associated with significant improvements in nutritional status, with a transition toward a normal nutritional profile among older adults at risk of or affected by malnutrition (Spirgienė et al. [47]). The qualitative study by Frøyland Alne et al. [18] reported that the meal-sharing intervention with nursing students was described by participants as feasible and acceptable and was associated with subjective improvements in appetite, enjoyment of meals, and food intake, as well as psychosocial benefits, despite the absence of standardised nutritional outcome measures.

When the nutritional component was embedded within multidomain interventions, findings were more heterogeneous. In multicomponent nurse-led programmes (Jeong & Chang, [44]; Song & Boo, [38]; Kang et al., [39]; Xu et al., [35]; Xu et al., [34]), nutritional outcomes were often not significantly different between groups or limited to specific subdomains, whereas more consistent effects were observed for frailty, physical function and psychosocial outcomes.

- **Pillar 2—Physical activity and exercise support**

This pillar includes nurse-led programmes promoting physical activity, structured exercise (strength, balance, endurance), motivational coaching, community interventions and prehabilitation, including digital and remote models. Nursing interventions in primary care incorporating behavioural change techniques and objective feedback (pedometer/accelerometer) were associated with significant increases in daily steps and time spent in moderate-to-vigorous physical activity, with effects maintained up to 12 months (Harris et al. [30]). Consistent results also emerge from interventions based on motivational interviewing, which promote improved levels of physical activity and progression through the stages of behavioural change (Sönmez Sari & Kitiş [36]). In multi-component community programmes, nurse-led physical activity is associated with improvements in functional performance (e.g., TUG, muscle strength, functional capacity) and reduced levels of frailty (Kang et al. [39]; Song & Boo, [38]; Yeh et al. [49]). In isolated contexts or those with limited access to services, nurse-led home exercise does not always produce significant changes in physical performance, but it can still lead to improvements in ADL and quality of life (Akihiro et al. [46]). Some studies reported improvements in psycho-emotional outcomes, including reductions in depressive symptoms and improvements in quality of life and perceived well-being (Song & Boo, [38]). Finally, multimodal remote prehabilitation led by nurses, supported by apps and remote contact, was feasible and safe, with acceptable completion rates and variable adherence across components (Sato et al. [45]).

- **Pillar 3—Nursing vaccination coaching**

No primary empirical studies focusing on nurse-led vaccination coaching interventions were identified; therefore, no results could be mapped for Pillar 3.

- **Pillar 4—Frailty monitoring and prevention of functional decline**

This is the most widely represented pillar, comprising interventions focused on frailty screening and multidimensional assessment, Comprehensive Geriatric Assessment (CGA), personalised care planning, nursing case management, structured home follow-up and fall prevention strategies.

- **Electronic frailty screening and proactive nurse-led primary care interventions.**

Two cluster trials by Bleijenberg et al. reported that EMR-based frailty screening combined with proactive nurse-led assessment and care planning was associated with small differences in daily functioning compared with usual care, with no significant improvements in quality of life (Bleijenberg et al. [24]). From an economic perspective, frailty screening (with or without the additional nurse-led programme) showed a high probability of cost-effectiveness compared with usual care, largely driven by cost savings, while incremental QALY gains attributable to the nurse-led component were small (Bleijenberg et al. [28]).

- **Multifactorial CGA-based programmes with follow-up: mixed to null results in well-developed primary care.**

Cluster trials evaluating nurse-led multifactorial CGA-based care models with individualised care plans reported no significant differences compared with usual care in disability prevention, quality of life, falls, hospitalisations, or mortality at 12 months (Suijker et al. [31]). In the related economic evaluation, the intervention arm generated higher total costs and showed a low probability of cost-effectiveness (Suijker et al. [32]). Similarly, a low-intensity nurse-led health promotion model delivered through community consultation clinics did not produce significant improvements and, in populations with high baseline frailty, was associated with transitions toward worse health profiles—highlighting the challenges of achieving clinically meaningful change when intervention intensity is limited and baseline vulnerability is high (Marcus-Varwijk et al. [40]).

- **Falls, fear of falling, and disability trajectories.**

A home-based cognitive–behavioural programme delivered by community nurses significantly reduced fear of falling and activity avoidance, improved disability, and reduced indoor falls, while no significant differences were observed in the total number of falls (Dorresteijn et al. [29]).

- **Transitional care and intensive post-discharge home follow-up.**

The Transitional Care Bridge model reported non-significant differences in ADL dependence and mortality compared with usual care; the authors also reported implementation challenges and lower-than-planned home-visit intensity (van der Vlegel-Brouwer et al. [43]). An intensive, personalised, multicomponent post-discharge home intervention delivered by a specialist geriatric nurse reported improvements in delirium symptoms, cognitive function, and functional status (ADL/IADL) compared with usual care (Verloo et al. [33]).

- **Integrated home care models, service utilisation, and continuity of care.**

Integrated service models characterised by nursing case management and systematic follow-up were associated with reductions in healthcare utilisation outcomes. In very frail older adults, an Integrated Care at Home programme based on nurse case management was associated with significant reductions in emergency department visits and hospital days, alongside lower caregiver stress (Tam et al. [41]). In Italy, embedding community nurses within a proactive community/social programme was associated with reduced one-year hospitalisations, particularly among pre-frail and frail individuals, while mortality differences were not statistically significant (Terracciano et al. [48]). In the perioperative setting, a nurse-led preoperative CGA with structured transfer of recommendations to general practitioners reported improved information transfer and continuity indicators; however, functional outcomes remained non-significant and GP engagement remained limited (Ron et al. [42]).

## 4. Discussion

The present study did not directly investigate immunosenescence or specific immunological biomarkers; instead, it adopted a pragmatic, clinically oriented approach focusing on nurse-led interventions targeting clinical and functional outcomes commonly associated with immunosenescence-related vulnerability, including frailty, functional decline, physical inactivity, and malnutrition. The four-pillar framework adopted in this scoping review is grounded in geriatric and gerontological literature describing these domains as interrelated manifestations linked to immune ageing and inflammaging [50,51]. Moreover, poor adherence to preventive interventions and healthy lifestyle behaviors is common among frail older adults and is associated with increased vulnerability to immunological aging and reduced physiological resilience [52]. The aim of the review was to map community and primary care nurse-led preventive interventions targeting modifiable clinical and behavioural determinants associated with immune ageing, mainly across Pillars 1, 2, and 4, as no primary studies were identified for Pillar 3 (vaccination coaching). These interventions may plausibly contribute to improving immunosenescence-related clinical correlates through behavioural and functional pathways; however, causal inferences on biological immune mechanisms cannot be drawn, as no immunological biomarkers were assessed. This approach is consistent with recent systematic reviews showing that nurse-led interventions have a variable but potentially significant impact on physical and mental outcomes in pre-frail and frail individuals, even in the absence of direct biological measures [53]. The findings of the present study largely confirm the evidence emerging from recent systematic reviews (Kasa et al. [22]; Zheng et al. [53]) and ongoing trial protocols (Saunders et al. [54]), which identify multicomponent nurse-led interventions combining physical exercise, nutritional education, and psychosocial support as the most effective strategy for reducing frailty among community-dwelling pre-frail and frail individuals, with significant improvements in physical (frailty index, physical performance), nutritional, and mental outcomes (depression, social support). Similar benefits of combined and coordinated interventions have also been reported in the context of protected discharge models, with reductions in hospital readmissions and improved continuity of care (Azzellino et al. [55]). In particular, our framework, structured around comparable pillars (nutrition and immunonutrition-related support, physical activity, and functional decline prevention), aligns with the “function-based care” approaches described by Zheng et al. [53], which demonstrated effectiveness rates of 84% for physical outcomes and 92% for mental outcomes. These approaches showed greater effectiveness than personalized integrated care models, often based primarily on Comprehensive Geriatric Assessment (CGA), which have frequently reported limited or inconsistent effects on activities of daily living and mobility, as also noted by Song and Boo [38] and Kasa et al. [22]. However, compared with more intensive interventions conducted in Asian community or senior centre settings such as those by Ha and Park [56], which involved twice-weekly sessions over 12 weeks with periodic telephone support, and Ng et al. [14], which included structured components with an initial phase of supervised exercise followed by home-based training, the interventions examined in the present review, implemented within resource-limited primary community care settings, show more modest effects on grip strength and the Timed Up-and-Go (TUG) test. This pattern is consistent with European primary care evidence, where CGA-based nurse-led programmes have often shown limited or inconsistent effectiveness in preventing functional decline at 12 months, within a generally limited and heterogeneous body of primary care nursing interventions [57]. This finding is further supported by European cluster trials showing that CGA-based nurse-led programmes in primary care yield modest or uncertain clinical benefits, particularly for quality of life and daily functioning (Bleijenberg et al. [24]; Suijker et al. [31]). Ongoing European pragmatic cluster trials are currently evaluating the effectiveness of nurse-led CGA models in primary care [58]. The lack of impact on quality of life and health service utilization mirrors findings from trials by Godwin et al. [59] and Marcus-Varwijk et al. [40], which reported limited effects on HRQoL and care-related outcomes despite proactive nurse-led programmes. These findings suggest that changes in “hard” outcomes may require follow-up periods longer than six months and a more intensive, multidisciplinary integration of care. From a nutritional perspective, improvements in the Mini Nutritional Assessment (MNA) tend to be more modest and variable than physical–functional outcomes, reflecting literature in which nurse-led interventions show stronger effects on frailty, performance, and mobility, while nutritional outcomes are less consistently assessed. In particular, targeted low-threshold interventions based on nutritional counselling or education in real-world settings have demonstrated significant improvements in MNA scores even with limited resources (Spirgienė et al. [47]), while approaches centred on the relational dimension of meals have mainly reported perceived benefits in appetite and psychosocial well-being (Frøyland Alne et al. [18]). Psychosocial outcomes (e.g., depression and social support) emerge instead as a particularly promising area: in recent syntheses, “function-based care” interventions show a high proportion of positive effects on mental health outcomes (Zheng et al. [53]), and community-based multicomponent studies include measures such as the Geriatric Depression Scale (GDS) and the Medical Outcomes Study–Social Support Survey (MOS-SSS) (Song & Boo [38]), consistent with interventions integrating cognitive–behavioural components and relational nursing coaching (Dorresteijn et al. [29]). Within a context of still limited Italian and European literature on proactive primary care models, the findings of this scoping review suggest that district-level nursing interventions may produce sustainable effects on frailty and depressive symptoms even at moderate intensity (8–12 weeks). These effects appear more likely when interventions are supported by structured follow-up and enhanced, ongoing telephone coaching, consistent with the broader evidence supporting nurse-led remote follow-up and telemedicine-based care models [60]. However, as also highlighted by Pairada et al. [57], the scalability of such models requires systematic cost-effectiveness evaluations and the overcoming of organizational and systemic barriers, such as poor continuity with general practitioners [57,61]. Overall, these findings highlight the pivotal role of community nurses in frailty prevention. From an implementation perspective, dedicated training and the adoption of standardized care frameworks (e.g., NANDA/NIC) **could represent** a pragmatic strategy to reduce methodological heterogeneity and facilitate integration between health and social care [61,62,63].

### 4.1. Limitations of the Study

This scoping review has some limitations. First, as a scoping review, it aimed to map the breadth of available evidence rather than to evaluate intervention effectiveness or establish causality. Second, none of the included primary studies assessed biological or immunological markers of immunosenescence; therefore, potential links with immunosenescence can only be discussed indirectly through clinical and functional correlates (e.g., frailty, physical function, and well-being). Third, the search was restricted to English-language studies published within the last 10 years, which may have excluded relevant evidence from other languages or earlier studies. Grey literature was not included, which may have limited the identification of locally implemented or non-indexed interventions. Finally, variability in intervention components, settings, and outcome measures limits direct comparability across studies, and the findings should therefore be interpreted with caution.

### 4.2. Implications for Clinical Practice

The findings of this scoping review suggest that community and primary care nurses may benefit from prioritising multicomponent preventive interventions integrating physical activity, nutritional counselling, and frailty monitoring, delivered with adequate intensity and continuity. Structured programmes with ongoing follow-up tended to show more consistent benefits on frailty, functional outcomes, and psychosocial well-being than low-intensity or single-contact approaches, particularly in higher-risk older adults. These results underscore the value of structured protocols and closer integration between nursing services, general practitioners, and social care to enhance continuity and scalability of preventive interventions. The absence of primary studies on nurse-led vaccination coaching also highlights an important opportunity for future nursing practice and research. The proposed NURSE AGE Framework (Nutrition counselling, Upgrading physical activity adherence, Risk assessment for frailty, Support for vaccination, Engagement of caregivers) provides a pragmatic synthesis of these priorities and supports the future development of structured nurse-led preventive pathways, including vaccination-related components. Future primary studies are needed to evaluate nurse-led vaccination coaching interventions in community and primary care settings and their potential impact on clinical outcomes in older adults.

## 5. Conclusions

This scoping review highlights the important role of community and primary care nurses in preventing frailty and functional decline in older adults through interventions addressing key clinical and functional determinants commonly associated with immunosenescence-related vulnerability. The available evidence suggests that structured, multicomponent nursing programmes of adequate intensity—particularly those integrating physical activity, nutritional support, and psychosocial components—tend to be associated with more consistent benefits than fragmented or low-intensity preventive approaches. The findings also indicate that frailty identification, when not accompanied by continuous and integrated interventions, is often associated with limited clinical impact. In this context, proactive nursing models characterised by continuity of care and integration with general practitioners and social care services appear to be associated with more favourable outcomes, especially in community and home care settings. With regard to vaccination-related nursing interventions, the available evidence remains limited, indicating an area that warrants further investigation. Similarly, current studies primarily focus on clinical and functional outcomes, with limited incorporation of immunological or biological markers. Future research should therefore evaluate nurse-led preventive interventions that include vaccination-related components and integrate immunological biomarkers, alongside clinical and functional outcomes, in order to better clarify potential links between nursing interventions, clinical outcomes, and immunosenescence-related vulnerability.

## Figures and Tables

**Table 1:** Characteristics of the included studies and summary of nurse-led interventions and main findings.

Author/Year	Study Design	Care Setting	Reference Conceptual Pillar(s)	Description of the Nurse-Led or Nurse-Delivered Nursing Intervention	Main Outcomes	Key Findings
**Bleijenberg et al., 2017 [28]**	Cluster-randomized controlled trial	Primary care/community (general practices, home-based assessment)	Pillar 4—Frailty monitoring and prevention of functional decline	Following electronic frailty screening based on routine EMR data, trained registered practice nurses conducted home-based Comprehensive Geriatric Assessments in frail older adults, developed individualized evidence-based care plans targeting geriatric syndromes, coordinated care with general practitioners, and provided proactive follow-up tailored to patients’ care needs.	Cost-effectiveness (QALYs, healthcare and informal care costs), health-related quality of life (EQ-5D), healthcare utilization	Both frailty screening alone and frailty screening plus nurse-led care showed a high probability of being cost-effective compared with usual care, mainly due to cost savings. The nurse-led proactive care program achieved slightly higher QALYs but added limited incremental value compared with screening alone, suggesting modest clinical benefits.
**Bleijenberg et al., 2016 [24]**	Cluster randomized controlled trial	Primary care, 39 general practices, community-dwelling individuals, Netherlands (Utrecht region)	Pillar 4—Frailty monitoring and prevention of functional decline	Following EMR-based frailty screening, practice nurses delivered a personalised programme including GFI assessment, home-based CGA, individualised care plans, care coordination with GPs, and tailored follow-up.	Daily functioning (modified Katz-15). Secondary outcomes: QoL (RAND-36, EQ-5D), healthcare utilisation, nursing home/assisted-living admission, mortality.	Participants who received the frailty screening intervention, with or without the nursing care program, showed less decline in functioning in daily activities at 12 months compared to those who received usual care; however, differences were modest and of uncertain clinical significance, with no significant benefits on quality of life.
**Dorresteijn et al., 2016 [29]**	Randomised controlled trial (RCT) with two groups and follow-up at 5 and 12 months	Home/community care (community-dwelling frail older adults), Netherlands	Pillar 2—physical activity and exercise support; Pillar 4—Frailty monitoring and prevention of functional decline	Home-based cognitive–behavioural programme led by community nurses (three home visits and four telephone contacts) to reduce fear of falling through cognitive restructuring, increasing self-efficacy, personalised goal setting, action planning, promoting safe behaviours and gradual exposure, with structured educational support and nursing follow-up.	Concerns about falls (FES-I). Secondary outcomes: activity avoidance (FES-IAB), disability (GARS—ADL/IADL), and falls (total and indoor).	The home nursing programme significantly reduced fear of falling and avoidance of activities at 5 and 12 months compared to usual care, improved disability and reduced indoor falls, with no significant differences in the total number of falls; effects were small to moderate but clinically relevant in a frail population.
**Frøyland Alne et al., 2021 [18]**	Qualitative exploratory–descriptive study (semi-structured interviews), 9 weeks, 23 student–elder pairs	Home/community care (elderly people living alone and receiving home care)	Pillar 1—nursing nutrition and immunonutrition support; Pillar 4—Frailty monitoring and prevention of functional decline	Low-threshold meal-sharing intervention: nursing students shared 1–2 meals/week for 9 weeks with older adults at risk of malnutrition, supporting meal preparation/presentation, meal environment adaptation, food fortification and intake, with informal nutritional observation and collaboration with home care nurses to personalise nutritional care.	Perceived experiences related to appetite, enjoyment of meals, food intake, body weight, psychological and social well-being, as well as facilitators and barriers to meal-sharing interventions.	Sharing meals with nursing students was perceived as a sustainable, low-cost nutritional intervention, associated with improved appetite, enjoyment of meals and food intake in older adults living alone, and promoted social and emotional well-being and better individualisation of nutritional care, despite the absence of standardised quantitative measures of nutritional outcomes.
**Harris et al., 2015 [30]**	Randomised controlled cluster trial (cluster RCT) with follow-up at 3 and 12 months	Primary care/local healthcare (general medical clinics)	Pillar 2—physical activity and exercise support	Complex nurse-led intervention delivered by general practice nurses, consisting of four individual consultations over three months on physical activity, integrating behavioural change techniques (goal setting, self-monitoring, increased self-efficacy), personalised feedback via pedometer/accelerometer, an individual walking plan, a physical activity diary and a structured support manual, with visual feedback and gradual increase in moderate-intensity walking.	Primary outcome: change in average daily number of steps measured objectively with an accelerometer at 3 months. Secondary outcomes: weekly time spent in moderate–vigorous physical activity (MVPA), MVPA in bouts ≥ 10 min, accelerometer counts, as well as adverse events, pain, mood, and anthropometric measurements, assessed at 3 and 12 months.	The nursing intervention significantly increased daily steps and time spent in MVPA compared to usual care at 3 and 12 months, with most activity in bouts of ≥ 10 min; no significant differences were found in adverse events, pain or mood. The intervention was well accepted, supporting the feasibility and effectiveness of nurse-led physical activity programmes in primary care.
**Jeong & Chang, 2025 [44]**	Randomised controlled trial, 2 groups, 12 weeks	Community senior centre (Seoul, Korea), women aged ≥ 65 years who are prefrail (K-FRAIL 1-2)	Pillar 1—nursing nutrition and immunonutrition support; Pillar 2—physical activity and exercise support; Pillar 4—frailty monitoring and prevention of functional decline	A 12-week multicomponent nurse-led health coaching programme (N-POWER; CARES model) including once-weekly group sessions (exercise: resistance/balance/flexibility; nutrition education; cognitive training) and once-weekly individual telephone coaching focused on personalised goal setting, self-monitoring, barriers/facilitators, and motivational support.	Primary outcome: frailty status measured using the K-FRAIL scale. Secondary outcomes: grip strength, physical function (SPPB), nutritional status (NQ-E), depressive symptoms (GDSSF-K) and cognitive function (CIST), assessed at baseline and at the end of the intervention.	The N-POWER programme prevented frailty progression in prefrail older women vs. usual care, with 33.3% improving from prefrailty to robustness, and improved grip strength, physical and cognitive function, and depressive symptoms; total nutritional status did not differ, although dietary balance improved.
**Kang et al., 2025 [39]**	Single-group pretest–posttest quasi-experimental study	Community setting (13 counties, Seoul district/public health center-based programme)	Pillar 1—nursing nutrition and immunonutrition support; Pillar 2—physical activity and exercise support; Pillar 4—Frailty monitoring and prevention of functional decline	An 8-week nurse-led, multifactorial group-based frailty prevention programme with weekly 90 min sessions (30 min health education on nutrition, oral health, chronic disease management, dementia prevention and mental health; 60 min structured exercise for strength, balance, flexibility and cardiorespiratory endurance), planned and adapted by public health nurses with monitoring, motivational support, adherence promotion and safety.	Functional capacity (Timed Up-and-Go test and grip strength), frailty status (frailty assessment tool used by home care services), metabolic health indicators (BMI, blood pressure, blood glucose).	The nursing intervention significantly improved functional capacity (reduced TUG time and increased grip strength) and reduced frailty levels (continuous score and categorical classification); among metabolic indicators, only blood glucose improved. Despite no control group, results suggest multifactorial nurse-led group programmes are feasible and effective in improving frailty and physical function in community-dwelling older adults.
**Kasa et al., 2024 [21]**	Quasi-experimental single-group study with pre-test, post-test and 12-week follow-up design	Community/local support (elderly people living at home in Bahir Dar, Ethiopia)	Pillar 4—Frailty monitoring and prevention of functional decline	Multidimensional nurse-led home-based intervention delivered by community nurses for 6 months, including six face-to-face educational sessions (ageing, healthy nutrition, physical activity, mental health, social interaction/support), a culturally adapted handbook and fortnightly telephone follow-ups, providing structured education, personalised counselling, motivational support and monitoring of physical, psychological and social needs.	Primary outcome: frailty status measured using the Tilburg Frailty Indicator—Amharic version (TFI-AM). Secondary outcomes: activities of daily living (Katz-ADL), nutritional status (Mini Nutritional Assessment and SNAQ), depressive symptoms (GDS-15) and quality of life (WHOQOL-BREF), assessed at baseline, at the end of the intervention and at 12 weeks of follow-up.	The nursing intervention significantly reduced frailty and depressive symptoms and improved nutritional status, ADL and overall quality of life; effects on frailty and nutrition were sustained at 12-week follow-up, while improvements in some quality-of-life dimensions and depression were not fully maintained over time.
**Lee et al., 2025 [37]**	Mixed-methods pragmatic quasi-experimental trial (pre–post with control group)	Community/local services (community service centres, Seoul, South Korea)	Pillar 2—physical activity and exercise support; Pillar 4—Frailty monitoring and prevention of functional decline (frailty prevention)	A 12-week nurse-led community-based frailty prevention programme (NurFP) including weekly individual nursing counselling, multidisciplinary group education (nutrition, self-management of chronic conditions, health), structured exercise (strength, balance, functional mobility) and weekly guided group walking sessions, coordinated by community nurses with self-monitoring diaries, personalised feedback, social support and involvement of community resources.	Primary outcome: frailty level measured using the Tilburg Frailty Indicator (TFI). Secondary outcomes: multilevel risk factors for frailty, including adherence to physical activity, healthy eating behaviours, self-management of chronic diseases, perceived social support, and perception of a community environment conducive to frailty prevention.	The NurFP programme significantly reduced overall frailty compared to controls, improving physical and social domains, and increased adherence to physical activity, self-management of chronic conditions and perceived social support. Qualitative findings highlighted a strengthened community environment and the central role of nurses as leaders and facilitators of sustainable local interventions.
**Marcus-Varwijk et al., 2020 [40]**	Quasi-experimental study	Community/local assistance (Community Health Consultation Offices for Seniors—Netherlands)	Pillar 1—nursing nutrition and immunonutrition support; Pillar 2—physical activity and exercise support; Pillar 4—Frailty monitoring and prevention of functional decline	Nurse-led health promotion and prevention intervention (Community Health Consultation Offices for Seniors, CHCO) for older adults ≥ 60 at risk of frailty and/or with unhealthy behaviours, including structured clinic/home consultations with multidimensional assessment, biometric measurements (BMI, blood pressure, blood sugar), personalised counselling using motivational interviewing, shared goal setting, preventive recommendations and referral when needed, with initial consultation, optional 3-month follow-up and 12-month reassessment.	Health outcomes: self-perceived health status, falls and fractures, biometric measurements (blood pressure, blood sugar, BMI, waist circumference) and health behaviours (smoking, alcohol consumption, balanced diet, physical activity). Care needs outcomes: frailty (Groningen Frailty Indicator) and progression of care needs, assessed through transitions in health profiles based on frailty and complexity (IM-E-SA).	The CHCO intervention showed no significant improvements in health outcomes or stabilisation of care needs versus usual care at 12 months; health behaviours and biometric parameters remained largely stable. More participants transitioned to worse health profiles, likely due to high baseline frailty, highlighting the difficulty of achieving clinically relevant changes with low-intensity nurse-led interventions in high-risk older adults.
**Ron et al., 2025 [42]**	Prospective observational study with parallel group design (non-randomised case–control)	Acute care hospital + primary care (hospital–community continuity, Israel)	Pillar 4—Frailty monitoring and prevention of functional decline	Pre-operative nurse-led geriatric assessment using CGA and the Assuta Frailty Score (functional, cognitive, nutritional and clinical domains), including structured frailty screening, risk identification for functional decline and personalised recommendations; assessment results and follow-up recommendations were proactively transferred to the general practitioner in the intervention group, while information transfer was left to the patient/caregiver in controls.	Functioning in daily activities (Barthel Index—ADL; Lawton IADL), physical and mental health status (SF-12), self-assessment of health status (scale 0–100), indicators of continuity of care and communication with the general practitioner, assessed 3 months after surgery.	Patients receiving the structured continuity of care model showed less functional decline in ADL/IADL and greater improvement in self-rated health versus usual care, although differences were not statistically significant. Information transfer to general practitioners was significantly higher, but GP involvement remained limited in both groups, highlighting systemic barriers to continuity of care.
**Sönmez Sari & Kitiş, 2024 [36]**	Randomised controlled trial (RCT)	Family Health Centre (Turkey)	Pillar 2—physical activity and exercise support	Nurse-led motivational interviewing intervention based on the Trans-Theoretical Model, delivered over 24 weeks to promote physical activity in older adults through regular nurse-conducted motivational interviews supporting behavioural change; the control group received usual care at the family care centre.	Level of physical activity measured using the Physical Activity Scale for the Elderly, step count using a pedometer, stages of behavioural change (Trans-Theoretical Model), Exercise Processes of Change Scale, Exercise Self-Efficacy Scale, Decisional Balance Scale for Exercise.	At the end of the intervention, the experimental group significantly improved physical activity behaviour, increasing steps and Physical Activity Scale for the Elderly scores versus controls, with significant progression in stages of change and a greater transition to the action stage.
**Sato et al., 2025 [45]**	Single-arm prospective intervention	Home-based remote (smartphone apps), Nara Medical University Hospital, Japan	Pillar 1—nursing nutrition and immunonutrition support; Pillar 2—physical activity and exercise support	Nurse-led remote multimodal prehabilitation programme, fully home-based and delivered via smartphone apps by perianesthesia nurses, including nutritional therapy (nutritional assessment and dietary counselling on calorie/protein intake), exercise therapy (smartphone-monitored walking and resistance exercises) and cognitive training; nurses coordinated the programme, provided instructions, monitored adherence and maintained regular contact by telephone/email during the preoperative period.	Primary outcome: programme feasibility (participation rate, adherence and completion). Secondary outcomes: incidence of post-operative delirium, post-operative complications (Clavien–Dindo ≥ III) and changes in functional disability assessed with WHODAS 2.0 in the perioperative period.	The remote multimodal prehabilitation programme led by peri-anaesthetic nurses was feasible and safe (50% participation, 92% completion) with no adverse events; low postoperative delirium (4.3%) and major complications (6.5%) were observed, supporting the implementation of remote nurse-led approaches in older surgical candidates to overcome hospital access barriers.
**Akihiro et al., 2018 [46]**	Controlled interventional study (non-randomised)	Home-based, isolated doctor-less island (Kuroshima, Kagoshima, Japan)	Pillar 2—physical activity and exercise support	A nurse-led home exercise programme, conducted over 3 months, aimed at elderly people living on an isolated island with no regular medical services. The intervention involved home exercises performed several times a week, including stretching, muscle strengthening, balance exercises and walking. The exercises were taught and supervised by public health nurses, trained by rehabilitation professionals, with regular home visits to support adherence and monitor physical condition.	Physical performance, functioning in activities of daily living (Functional Independence Measure—FIM) and health-related quality of life (SF-36), assessed at baseline and after 3 months.	No significant differences in physical performance were found between groups; however, the intervention group showed significant improvements in ADL (FIM) and several quality-of-life domains (physical and mental), while controls tended to worsen. The findings suggest nurse-led home exercise programmes are feasible and potentially effective in improving autonomy and quality of life in older adults living in isolated settings.
**Song & Boo, 2022 [38]**	Quasi-experimental (non-equivalent control)	Community setting/public health centres and senior centres (South Korea); community-dwelling prefrail or frail older adults living alone	Pillar 1—nursing nutrition and immunonutrition support; Pillar 2—physical activity and exercise support	Nurse-led multicomponent community-based group intervention lasting 12 weeks, including weekly physical exercise (stretching, resistance with elastic bands, aerobic movements), cognitive training and social activities, and monthly education on nutrition and chronic disease management; nurses coordinated the programme, supported participation, monitored safety and facilitated continuity of care with local physicians.	Frailty index, TUG, handgrip, depression (GDS-SF-K), social activity, social support (MOS-SSS)	The nurse-led multicomponent intervention significantly improved frailty, mobility (TUG), depressive symptoms, social activity and social support versus controls, with sustained psychosocial and mobility benefits at follow-up; handgrip strength showed no significant between-group differences. Findings suggest multicomponent nurse-led community interventions can improve physical and psychosocial functioning in prefrail/frail older adults living alone.
**Spirgienė et al., 2018 [47]**	cross-sectional descriptive study with a 6-month follow-up after nurse-delivered nutritional recommendations	Rural primary health care centre, Lithuania	Pillar 1—nursing nutrition and immunonutrition support	Nurse-led nutritional education for older adults at risk of malnutrition/malnourished, including structured nutritional assessment using the Mini Nutritional Assessment (MNA), written nutritional recommendations developed by a geriatrician and explained by nurses during outpatient/home visits, education on healthy eating and malnutrition prevention, and monthly nurse telephone follow-ups to support adherence and address doubts.	Nutritional status measured using the Mini Nutritional Assessment (MNA); prevalence of malnutrition and risk of malnutrition; change in MNA score after 6 months in participants at risk or malnourished.	About half of older adults in the rural community were at risk of malnutrition/malnourished. Among those who followed recommendations, MNA scores improved significantly at 6 months with many transitioning to normal nutritional status, suggesting low-intensity nurse-led nutrition education can improve nutritional status in rural older adults.
**Suijker et al., 2016 [31]**	Cluster randomized controlled trial	Primary care/community (Netherlands); elderly people aged ≥ 70 years, community, at risk of functional decline (ISAR-PC ≥ 2)	Pillar 4—Frailty monitoring and prevention of functional decline	Multifactorial nurse-led intervention coordinated by Community Care Registered Nurses, including home-based CGA followed by an individualised Care and Treatment Plan with evidence-based multifactorial interventions, multiple follow-up home visits, discussion of CGA results with the general practitioner, and coordinated care (case management, self-management support, patient-centred care) with other professionals as needed.	Primary outcome: functional disability measured using the modified Katz-ADL index (0–15) at 12 months. Secondary outcomes: quality of life (EQ-5D), emotional well-being (RAND-36), perceived quality of life, use of health services (hospitalisations, out-of-hours visits), falls and mortality.	After 12 months, the multifactorial nurse-led programme showed no significant benefits versus usual care in preventing functional decline and no effects on quality of life, hospitalisations, falls or mortality, suggesting no superiority over standard care in a well-developed primary care setting.
**Suijker et al., 2017 [32]**	Cluster randomized trial (24 practices)	Primary care/community (general practice, Netherlands); older adults ≥ 70 years at risk of functional decline	Pillar 4—Frailty monitoring and prevention of functional decline	Multifactorial nurse-led intervention coordinated by community care registered nurses, based on a comprehensive geriatric assessment (CGA) and personalised interventions tailored to individual needs. Nurses coordinated care, developed individualised care plans, and conducted multiple follow-up home visits to identify geriatric problems early and support continuity of care, in addition to the usual care provided by general practitioners.	Functional disability measured using the modified Katz Activities of Daily Living (ADL) index and Quality-Adjusted Life Years (QALYs), assessed during a 12-month follow-up; direct healthcare costs analysed from a healthcare system perspective.	No statistically significant differences were observed in disability (Katz-ADL) or QALYs at 12 months; total costs were significantly higher in the intervention group, resulting in a low probability of cost-effectiveness. The authors concluded that the multifactorial nursing care model was not cost-effective in preventing or delaying new disabilities during the observation period.
**Tam et al., 2025 [41]**	Retrospective observational	Home/community care (Integrated Care at Home programme, Hong Kong)	Pillar 4—Frailty monitoring and prevention of functional decline	Integrated Care at Home (ICAH) programme for very frail older adults based on nursing case management, assigning a community nurse case manager for regular home visits, nursing follow-ups, ad hoc telephone consultations and coordination with geriatricians, including CGA, continuous clinical/functional monitoring, wound and medication management, support for medical devices, caregiver education/support, advance care planning and coordination of hospital-to-community transitions.	Use of hospital services: visits to the Emergency Department (ED) and days spent in hospital in the 180 days before and after enrolment in the programme; caregiver stress measured using the Relative Stress Scale (RSS) at 3 months.	After ICAH enrolment, emergency room visits and hospitalisation days significantly decreased (15.3 to 3.2 and 266.4 to 29.7 per 1000 patient-days, respectively), and caregiver stress decreased at 3 months, suggesting integrated home-based models with strong nursing case management can improve continuity of care and reduce hospital burden in very frail older adults.
**Terracciano et al., 2021 [48]**	Nested case–control study (community nurse integrated proactive care programme)	Community/integrated local support (Long Live the Elderly! programme, Rome and Naples, Italy)	Pillar 4—Frailty monitoring and prevention of functional decline; Pillar 1—Nursing nutrition and immunonutrition support (limited)	Integration of community nurses into a proactive social programme (“Long Live the Elderly!”), including multidimensional frailty assessment using the Functional Geriatric Evaluation and personalised interventions with social services/other professionals (home fall prevention, nutritional education and MNA assessment, medication review, adherence support, socialisation, psychosocial support and coordination of local care).	Primary outcomes: 1-year mortality and 1-year hospitalisation rate, standardised for age, sex and frailty level. Secondary outcomes: frailty level and distribution of risk classes.	Integration of community nurses into the proactive social programme reduced 1-year hospitalisation rates versus standard care (10.8% vs. 15.4%), while mortality differences were not statistically significant; reductions were greater in pre-frail and frail individuals. Findings suggest multidimensional frailty assessment and proactive nurse-led care integrated with social interventions may reduce hospital use in older adults >75 years.
**van der Vlegel-Brouwer et al., 2023 [43]**	Pre–post cohort study (quantitative phase of a mixed-methods study)	Post-hospital transitional care, Netherlands	Pillar 4—Frailty monitoring and prevention of functional decline	Transitional Care Bridge (TCB), a nurse-led transitional care programme initiated in hospital and continued at home, including ISAR-HP screening in the ED, ward-based CGA, structured handover, and community nurse home visits within 48 h post-discharge and at 2, 6, 12 and 24 weeks to monitor clinical/functional status, ADL, delirium/falls/malnutrition risk, support therapy management, social participation and caregivers, and coordinate care with GPs and local services.	Functional decline (dependence in ADL—Katz Index), mortality at 1 and 3 months, use of health services (readmissions, ED visits, GP visits), risk of delirium, risk of malnutrition, self-assessment of health and quality of life, measured up to 3 months after discharge.	At 3 months, the TCB group showed a lower proportion of ADL dependence versus usual care (36.7% vs. 47.1%), but differences were not statistically significant; no significant differences were found in health service use or other outcomes, and mortality was lower but not significant. Implementation problems and low adherence (fewer visits than expected) may have influenced results.
**Verloo et al., 2015 [33]**	RCT pilot	Home/community care (Switzerland); elderly people aged ≥ 65 discharged from hospital with a home care prescription	Pillar 4—Frailty monitoring and prevention of functional decline; Pillar 1—Nursing nutrition and immunonutrition support (secondary); Pillar 2—physical activity and exercise support)	Nurse-led multicomponent personalised post-discharge intervention delivered by a specialist geriatric nurse during five home visits (48 h, 72 h, 7, 14 and 21 days), based on the Neuman System Model and Intervention Mapping, using 15 nursing protocols across six domains, including delirium identification/monitoring (CAM) and cognitive orientation, ADL/IADL support, dehydration/constipation prevention, mobility promotion, infection/fall prevention, medication review, nutritional education, sleep support, social network strengthening and caregiver support, tailored to individual risks.	Symptoms of delirium (Confusion Assessment Method—CAM); cognitive function (Mini-Mental State Examination—MMSE); functional status (Katz ADL + Lawton IADL), assessed at baseline (M1) and after 1 month (M2).	After adjustment for age, comorbidity, polypharmacy and cognitive impairment, the intervention group showed significant reductions in delirium symptoms and improvements in cognitive function and ADL/IADL versus usual care; the intervention was feasible, safe and well accepted. Findings suggest multicomponent personalised home-based nursing interventions may help prevent cognitive and functional decline post-discharge.
**Xu et al., 2020 [34]**	Pilot RCT, 3 arms, open-label, 6 months	Primary care and community settings (Hong Kong); adults ≥ 60 years with Mild Cognitive Impairment (MCI)	Pillar 1—Nursing nutrition and immunonutrition support; Pillar 2—physical activity and exercise support; Pillar 4—Frailty monitoring and prevention of functional decline	Nurse-led risk factor modification (RFM) intervention delivered at baseline, 3 and 6 months using motivational interviewing (FRAMES), alone or combined with Tai Chi (3×/week for 12 weeks) and group cognitive training (CPR), including personalised nutritional intervention, monitoring/management of metabolic and vascular risk factors, and counselling on physical activity, smoking and therapeutic adherence.	Primary outcome: feasibility (recruitment rate, adherence, dropout). Secondary outcomes: cognitive function (HK-MoCA, ADAS-Cog, CDR), functional disability (DAD), quality of life (EQ-5D), depression (GDS-15), anxiety (GAS-20), physical activity (PASE), use of health services and diet (food frequency questionnaire).	The study demonstrated feasibility with high adherence to RFM (>85%); the RFM group improved cognitive function (HK-MoCA), while the CPR group improved functional disability (DAD), reduced anxiety (GAS-20) and reduced healthcare costs. Findings suggest multidomain nurse-led interventions are feasible and potentially effective in slowing cognitive decline in older adults with MCI in primary care.
**Xu et al., 2025 [35]**	Three-arm, open-label, blinded-endpoint randomised controlled trial with 15-month follow-up	Primary care & community setting (Lek Yuen Health Centre, Hong Kong); adults aged 60–80 years with Mild Cognitive Impairment (HK-MoCA 19–25)	Pillar 1—Nursing nutrition and immunonutrition support; Pillar 2—physical activity and exercise support; Pillar 4—Frailty monitoring and prevention of functional decline	Nurse-led Risk Factor Modification (RFM) delivered every 3 months using motivational interviewing (FRAMES) to modify dementia-related risk factors, including personalised dietary counselling, counselling on physical activity, smoking cessation and medication adherence, and monitoring of vascular/metabolic risk factors (BMI, blood pressure, glucose, lipids); in the CPR group, RFM was combined with Tai Chi (3×/week for 12 weeks) and group-based cognitive training in the first 3 months.	Primary outcome: cognitive function measured by ADAS-Cog Z score at 15 months. Secondary outcomes: cognitive and functional status (CDR-SOB, DAD), quality of life (EQ-5D, EQ-VAS), depressive symptoms (GDS-15), anxiety (GAS-20), physical activity (PASE), dietary adherence score, alcohol consumption (AUDIT-C), and health service utilisation.	No significant between-group differences were observed in ADAS-Cog or secondary outcomes at 6, 12 or 15 months, while all groups improved over time. Per-protocol/subgroup analyses suggested short-term benefits of the multicomponent CPR intervention in participants with hypertension or depressive symptoms and improved dietary adherence at 12 months; overall, nurse-led RFM was not superior to health advice, indicating the need for refined and longer multidomain interventions in high-risk subgroups.
**Yeh et al., 2022 [49]**	Quasi-experimental single-group pre–post-test pilot study	Rural community/community activity centres (Yunlin County, Taiwan)	Pillar 2—physical activity and exercise support; Pillar 4—Frailty monitoring and prevention of functional decline	Innovative Summer Camp (ISC), a nurse-led 12-week health promotion programme coordinated with Community Care Workers, including daily 90 min activities (5 days/week) based on modified Baduanjin exercises, recreational breathing games, recreational motor activities (table tennis) and singing (karaoke); nurses designed the programme, trained CCWs, monitored vital signs/blood pressure, ensured safety and supervised adherence.	Physiological biomarkers (weight, waist circumference, blood pressure, lung function parameters: FVC, predicted FVC%, predicted FEV1%) and health-related fitness (muscle strength and endurance, static and dynamic balance, cardiorespiratory capacity), assessed at baseline and after 12 weeks (post-intervention).	After 12 weeks, the ISC programme significantly improved physiological biomarkers (reduced weight, waist circumference and systolic blood pressure; improved lung function) and physical/functional fitness (muscle strength, balance, cardiorespiratory capacity), with high participant satisfaction and well-being, suggesting low-intensity low-cost nurse-led community programmes can improve physical capacity and well-being in rural older adults.

## Data Availability

All evidence supporting the conclusions of this work originates from published sources, which are referenced throughout the manuscript.

## Supplementary Materials

The following supporting information can be downloaded at: https://www.mdpi.com/article/10.3390/medicina62020262/s1, **Search strategy and full search strings**.
