# Supplementary material for: Nurse-Led Interventions Targeting Clinical Correlates of Immunosenescence in Older Adults: A Scoping Review
Source: Medicina (Kaunas). 2026 Jan 26;62(2):262. doi: 10.3390/medicina62020262 (PMC12941727; doi:10.3390/medicina62020262)
Supplement: Supplementary file 1 [file medicina-62-00262-s001.zip › medicina-4100675-supplementary.pdf]

Scopus

General string

(TITLE-ABS-KEY("community nursing" OR "home care nursing" OR "primary care nurse"

OR "nurse-led" OR "community nurse"))

AND

(TITLE-ABS-KEY("older adults" OR elderly OR "frail older" OR "aging population"))

AND

(TITLE-ABS-KEY(nutrition OR "diet" OR "micronutrients"

OR "physical activity" OR exercise

OR vaccination OR immunization

OR frailty OR "functional decline"))

AND NOT

(TITLE-ABS-KEY(pediatr* OR oncology OR cancer OR "intensive care"))

PILLAR 1

(TITLE-ABS-KEY("community nursing" OR "home care nursing" OR "nurse-led"))

AND

(TITLE-ABS-KEY(nutrition OR diet OR "nutritional status" OR "micronutrients" OR "vitamin D"))

AND

(TITLE-ABS-KEY("older adults" OR elderly OR "frail older"))

PILLAR 2

(TITLE-ABS-KEY("community nursing" OR "home care nurse" OR "nurse-led"))

AND

(TITLE-ABS-KEY("physical activity" OR exercise OR mobility OR "functional capacity"))

AND

(TITLE-ABS-KEY("older adults" OR elderly OR "frail older"))

PILLAR 3

(TITLE-ABS-KEY("community nurse" OR "primary care nurse" OR "nurse-led"))

AND

(TITLE-ABS-KEY(vaccination OR immunization OR "vaccine uptake"))

AND

(TITLE-ABS-KEY("older adults" OR elderly OR "frail older"))

PILLAR 4

(TITLE-ABS-KEY("community nurse" OR "home care nurse" OR "nurse-led"))

AND

(TITLE-ABS-KEY(frailty OR "frailty assessment" OR "functional decline" OR sarcopenia))

AND

(TITLE-ABS-KEY("older adults" OR elderly OR "frail older"))

Wos

General string

TS=("community nursing" OR "home care nursing" OR "primary care nurse" OR "nurse-led" OR "community nurse")

AND

TS=("older adults" OR "elderly" OR "frail older" OR "aging population")

AND

TS=("nutrition" OR "diet" OR "micronutrients" OR "physical activity" OR "exercise"

OR "vaccination" OR "immunization" OR "frailty" OR "functional decline")

NOT

TS=("pediatric*" OR "oncology" OR "cancer" OR "intensive care")

PILLAR 1

TS=("community nursing" OR "home care nursing" OR "nurse-led")

AND

TS=(nutrition OR diet OR "nutritional status" OR "micronutrients" OR "vitamin D")

AND

TS=("older adults" OR elderly OR "frail older")

PILLAR 2

TS=("community nurse" OR "home care nursing" OR "nurse-led")

AND

TS=("physical activity" OR exercise OR mobility OR "functional capacity")

AND

TS=("older adults" OR elderly OR "frail older")

PILLAR 3

TS=("community nurse" OR "primary care nurse" OR "nurse-led")

AND

TS=(vaccination OR immunization OR "vaccine uptake")

AND

TS=("older adults" OR elderly OR "frail older")

PILLAR 4

TS=("community nurse" OR "home care nursing" OR "nurse-led")

AND

TS=(frailty OR "frailty assessment" OR "functional decline" OR sarcopenia)

AND

TS=("older adults" OR elderly OR "frail older")

Pubmed

General string

("community nursing"[Title/Abstract]

OR "nurse-led"[Title/Abstract]

OR "home care nursing"[Title/Abstract]

OR "primary care nurse"[Title/Abstract])

AND

("older adults"[Title/Abstract] OR "elderly"[Title/Abstract] OR "frail older"[Title/Abstract])

AND

("nutrition"[Title/Abstract] OR "physical activity"[Title/Abstract]

OR "exercise"[Title/Abstract] OR "vaccination"[Title/Abstract] OR "frailty"[Title/Abstract])

NOT

("pediatr*"[Title/Abstract] OR "intensive care"[Title/Abstract]

OR "oncology"[Title/Abstract] OR "cancer"[Title/Abstract])

PILLAR 1

("community nursing"[Title/Abstract]

OR "home care nursing"[Title/Abstract]

OR "primary care nurse"[Title/Abstract]

OR "nurse-led"[Title/Abstract])

AND

("older adults"[Title/Abstract] OR "elderly"[Title/Abstract] OR "frail older"[Title/Abstract])

AND

("nutrition"[Title/Abstract] OR "diet"[Title/Abstract]

OR "nutritional status"[Title/Abstract]

OR "micronutrients"[Title/Abstract] OR "vitamin D"[Title/Abstract]

OR "protein intake"[Title/Abstract])

NOT

("pediatr*"[Title/Abstract] OR "oncology"[Title/Abstract]

OR "cancer"[Title/Abstract] OR "intensive care"[Title/Abstract])

PILLAR 2

("community nursing"[Title/Abstract]

OR "home care nursing"[Title/Abstract]

OR "primary care nurse"[Title/Abstract]

OR "nurse-led"[Title/Abstract])

AND

("older adults"[Title/Abstract] OR "elderly"[Title/Abstract] OR "frail older"[Title/Abstract])

AND

("physical activity"[Title/Abstract] OR "exercise"[Title/Abstract]

OR "mobility"[Title/Abstract] OR "functional capacity"[Title/Abstract]

OR "mobility program"[Title/Abstract])

NOT

("pediatr*"[Title/Abstract] OR "oncology"[Title/Abstract]

OR "cancer"[Title/Abstract] OR "intensive care"[Title/Abstract])

PILLAR 3

("community nursing"[Title/Abstract]

OR "home care nursing"[Title/Abstract]

OR "primary care nurse"[Title/Abstract]

OR "nurse-led"[Title/Abstract])

AND

("older adults"[Title/Abstract] OR "elderly"[Title/Abstract] OR "frail older"[Title/Abstract])

AND

("vaccination"[Title/Abstract] OR "immunization"[Title/Abstract]

OR "vaccine uptake"[Title/Abstract] OR "vaccine adherence"[Title/Abstract])

NOT

("pediatr*"[Title/Abstract] OR "oncology"[Title/Abstract]

OR "cancer"[Title/Abstract] OR "intensive care"[Title/Abstract])

PILLAR 4

("community nursing"[Title/Abstract]

OR "home care nursing"[Title/Abstract]

OR "primary care nurse"[Title/Abstract]

OR "nurse-led"[Title/Abstract])

AND

("older adults"[Title/Abstract] OR "elderly"[Title/Abstract] OR "frail older"[Title/Abstract])

AND

("frailty"[Title/Abstract] OR "frailty assessment"[Title/Abstract]

OR "functional decline"[Title/Abstract] OR "sarcopenia"[Title/Abstract]

OR "performance status"[Title/Abstract])

NOT

("pediatr*"[Title/Abstract] OR "oncology"[Title/Abstract]

OR "cancer"[Title/Abstract] OR "intensive care"[Title/Abstract])

23/12/2025
